# Bacteriocins From LAB and Other Alternative Approaches for the Control of *Clostridium* and *Clostridiodes* Related Gastrointestinal Colitis

**DOI:** 10.3389/fbioe.2020.581778

**Published:** 2020-09-11

**Authors:** Svetoslav D. Todorov, Hye-Ji Kang, Iskra V. Ivanova, Wilhelm H. Holzapfel

**Affiliations:** ^1^Advanced Green Energy and Environment Institute (AGEE), Handong Global University, Pohang, South Korea; ^2^HEM Inc., Handong Global University, Pohang, South Korea; ^3^Department of General and Applied Microbiology, Faculty of Biology, Sofia University “St. Kliment Ohridski”, Sofia, Bulgaria

**Keywords:** *Clostridium*, *Clostridiodes*, bacteriocins, probiotics, biotherapeutics, lactic acid bacteria, gut microbiota, dysbiosis

## Abstract

The gut microbiome is considered as a promising target for future non-conventional therapeutic treatment of inflammatory and infectious diseases. The search for appropriate safe and beneficial (lactic acid bacterial and other) putative probiotic strains and/or their antimicrobial metabolites represents a challenging approach for combating several problematic and emerging infections. The process of selecting suitable strains, especially of lactic acid bacteria (LAB) with superior properties, has been accelerated and intensified during the past two decades, also thanks to recent developments in lab techniques. Currently, special focus is on the potential of antimicrobial metabolites produced by some LAB strains and their application as active therapeutic agents. The vision is to develop a scientific basis for ‘biotherapeutics’ as alternative to conventional approaches in both human and veterinary medicine. Consequently, innovative and promising applications of LAB to the therapeutic practice are presently emerging. An overview of the existing literature indicates that some antimicrobial metabolites such as bacteriocins, widely produced by different bacterial species including LAB, are promising biotherapeutic agents for controlling infections caused by potential pathogens, such as *Clostridium* and *Clostridiodes*. Non-conventional, safe and well designed therapeutic treatments may contribute to the improvement of gut dysbiotic conditions. Thereby gut homeostasis can be restored and inflammatory conditions such as gastrointestinal colitis ameliorated. Combining the knowledge on the production, characterization and application of bacteriocins from probiotic LAB, together with their antibacterial properties, appears to be a promising and novel approach in biotherapy. In this overview, different scenarios for the control of *Clostridium* spp. by application of bacteriocins as therapeutic agents, also in synergistic combination with antibiotics, will be discussed.

## Introduction

Since early human history the beneficial influence of fermented foods on the human gut has been appreciated; numerous ancient societies and cultures have consumed fermented foods such as yogurt as a therapy for treatment of diarrhea and other adverse gut conditions (Holzapfel, 2006). The development of microbiology as a discipline during the second half of the 19th century soon revealed the beneficial association of lactic acid bacteria (LAB) as a major microbial group associated with fermented foods. This era has also provided a scientific foundation for bacteriotherapy which has probably been pioneered by Döderlein (1892) when he reported on a vaginal Gram-positive “bacillus” with antagonistic activity against staphylococci, also suggesting lactic acid as underlying basis of this antagonism. Called “Döderlein’s bacillus,” later studies have focused on these (catalase-negative) ‘lacto-bacilli,’ their beneficial association with the human host and their role in balancing vaginal ecology (Lash and Kaplan, 1926; Cruickshank, 1931). A beneficial association of LAB with the human host and the intestinal tract was underlined by Metchnikoff (1908) when he proposed the high intake of fermented milk products to be related to longevity.

Developments in biological sciences have been fundamental, at least during the recent history of 200 years, for the development and accumulation of knowledge in clinical practices. Gut microbiota are considered as a promising target for future therapeutic treatment of inflammatory and infectious diseases (Mukherjee et al., 2018). Current biotherapy approaches involve the careful selection of appropriate strains and/or specific antimicrobial metabolites in order to meet exigencies when targeting specific (intestinal) pathogens such as some *Clostridium* and *Clostridiodes* spp. In the past two decades the search for LAB with probiotic properties and strains for application as active therapeutic agents has intensified. Special attention is currently directed at non-conventional “anti-infective” therapies, some of which involve specific vaccines (Czaplewski et al., 2016), antimicrobial metabolites such as antimicrobial peptides (Mahlapuu et al., 2016) and bacteriocins (Cotter et al., 2013; Hanchi et al., 2017; Chikindas et al., 2018), and also includes bacteriophage therapy and the application of predatory bacteria (Allen, 2017; Vieco-Saiz et al., 2019). Moreover, thanks to their adjuvant properties, LAB have been suggested for potential replacement of classical, attenuated microbial carriers that may frequently induce pathogenicity in the host (Szatraj et al., 2017). These examples represent feasible alternatives to current approaches, and show potential of application both in human and veterinary medicine.

With innovative applications of LAB in the therapeutic practice currently emerging, the focus on “natural” or “biological” approaches (as opposed to the use of antibiotics and chemical drugs) for combating infectious diseases have moved toward a major research area. In particular the search for functional LAB strains with potential application in human and veterinary medicine, also in combination with the production of beneficial (anti-pathogenic) bacteriocins and probiotic characteristics, is actively being pursued. The challenge of finding specific antimicrobial properties of produced antimicrobial metabolites beyond organic acids (Tejero-Sariñena et al., 2012) by LAB, concomitantly with desirable (probiotic) characteristics in a single strain remains an ultimate goal (Lerner et al., 2019). Nevertheless, several innovative therapeutic applications of LAB have emerged, and, as research reports are increasingly showing, LAB can be considered as promising and additional/alternative therapeutic agents for the control of some infectious diseases. With the rapid technological development in various branches of the life sciences and the accumulation of new knowledge in microbiology, physiology and medicine, important changes to long-established practices are now being introduced. Aside from the novel potentially-beneficial properties of candidate probiotic LAB strains, their safety assessment requires special attention (Sanders et al., 2010; Brodmann et al., 2017), with particular focus on the presence of known virulence factors, the production of biogenic amines and antibiotic resistance determinants. Numerous well-known and recently characterized virulence determinants are now mapped in members of the genera *Enterococcus* and *Streptococcus*. However, recently some of these virulence related determinants have also been detected in lactobacilli with previous Generally Recognized As Safe (GRAS) status; this could possibly signify potential hurdles for developers and manufacturers of probiotics and strains for use as bio-therapeutic agents. A risk of horizontal transfer of virulence factors has been identified when (probiotic) LAB and pathogenic strains colonize the same ecological niche. Uncontrolled or wrong therapeutic use of antibiotics and even bacteriocinogenic LAB strains may increase this risk, thereby underlining the importance of reliable safety assessment of LAB strains at the intraspecies level, before their application (Leuschner et al., 2010; Brodmann et al., 2017; Holzapfel et al., 2018). It is acknowledged that GRAS status cannot be applied to entire species or genera. Intraspecies diversity underpins the fact that strain differences can be related to the presence of potential virulence factors, previously not investigated in a particular bacterial species, or neither have been transformed via horizontal gene transfer as a result of inter-bacterial interaction. Safety aspects of beneficial cultures (probiotics and bacteriocinogenic bacteria) are a critical point in the approval and distribution of new probiotic cultures for human and animal applications. This process includes not only strict evaluation of the expression of virulence factors by physiological and biochemical approaches, but deep bio-molecular investigations for presence of genes related to virulence factors, also including antibiotic resistance. Antibiotic resistance in beneficial LAB may be a delicate point. Comprehensive risk assessment on the spread of resistant genes to human health has apparently not been conducted yet. Investigations on the behavior, adaptation and dynamics of probiotics under conditions representing the human gut, also accounting for the presence of antibiotics, therefore appear imperative (Zheng et al., 2017). In particular, probiotic cultures should be free of transferable genetic determinants related to antibiotic resistance in order to prevent their transfer to recipient strains. Special attention should be given to genes located on transmittable genetic elements, such as plasmids, transposons, and chaperons. Antibiotic resistance genes located on the bacterial chromosome are generally considered as a lower level of safety concern. However, every case needs to be evaluated on a strain specific basis also by considering the antibiotic specificity. On the other side, well defined antibiotic resistance of a probiotic culture might be considered as beneficial against the scope of its application in combination with a specific antibiotic. Thereby its synergetic interaction may be exploited by combining, e.g., an antibiotic and probiotic LAB strain under well controlled conditions.

Apart from the safety evaluation of viable bacterial strains, cytotoxicity of bacteriocins and other metabolites requires specific attention. Almost one century since the discovery of nisin its long history of safe food-associated use can be attributed to an extremely low cytotoxicity index similar to that of NaCl (Jozala et al., 2007), its sensitivity to digestive proteases, and the absence of any negative influence on the sensory properties of food (Pongtharangkul and Demirci, 2004). On other hand, some bacteriocins, including nisin, pediocin PA-1 and plantaricin A, appear to have elevated cytotoxicity against some kinds of cancer cells in comparison to normal cells. Altogether, application approval of a newly characterized bacteriocin should be supported by information on its cytotoxicity according to recommended accepted clinical standards.

**FIGURE 1:**
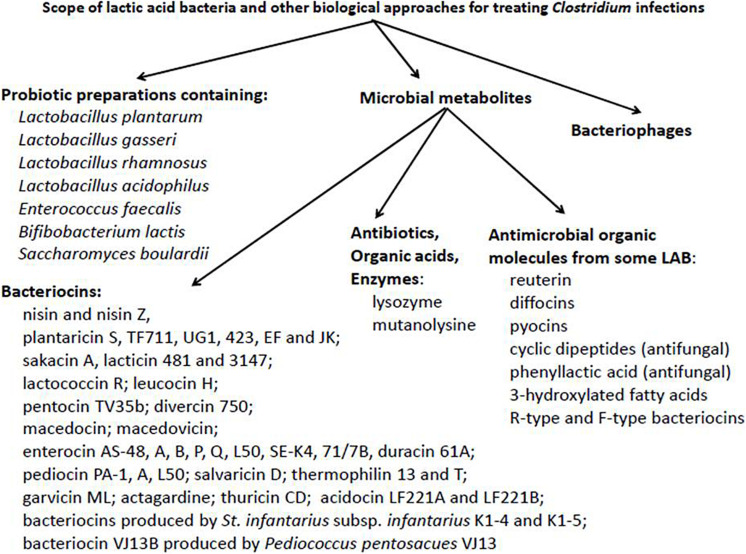
Potential scope of lactic acid bacteria and other non-conventional biological approaches for the treatment of *Clostridium* infections.

In this overview we are looking into the potential of biological approaches for non-conventional therapeutic treatment of inflammatory and infectious diseases. A particular focus will deal with the combating of *Clostridium* infections and the prospect of widening the applications of beneficial LAB and their metabolites for this purpose. Reference will also be made to the inhibition of heterogeneous target organisms, including pathogens associated with colitis. Information basic to potential biological approaches is summarized, at least in part, in Figure 1.

With this paper we attempt to find answers on questions related to, e.g.:

• Realistic and practical applications of LAB and their metabolites as tools of therapeutic adjuncts, but also including major limitations;
• A clear borderline between reality and fiction in these approaches;
• Whether some of the more significant issues have been overlooked in recent reports on previously-unexplored applications of bacteriocins;
• Possible applications of bacteriocin producers as potential probiotic cultures with the concomitant effective control of pathogens related to colitis;
• The purification level of antimicrobial metabolites such as antimicrobial peptides and considerations whether combinations with conventional antibiotics might be one way of optimization toward application.

## Bacteriocins

The beneficial practical application of antimicrobial peptides is not a novel concept. It may even be claimed that, for the centuries, bacteriocins and other antimicrobial metabolites have unknowingly been involved as natural preservatives when produced by LAB in traditional food fermentation processes. Nisin is the most well known, deepest researched, and commercially most widely used bacteriocin in food preservation, presently approved in at least 50 countries (Delves-Broughton et al., 1996). Its discovery in the 1930s was followed 30 years later by its first commercial introduction for food preservation in the United Kingdom. Since then the prospect of applying bacteriocins in food biopreservation has found general acceptance in food safety approaches (Abee et al., 1995; Silva et al., 2018).

Potential therapeutic application of bacteriocins and other antimicrobials produced by LAB in human and veterinary medicine represents a relatively new area of research investigations. It should be acknowledged that traditional medicine has recommended the application of some products resulting from (LAB associated) food fermentation processes as alternatives in the treatment of some diseases. Such practices are well known for the Maasai tribe of East Africa where the consumption of the traditional fermented milk product ‘*kule naoto*’ is considered to have therapeutic value for curing of and/or protection against ailments such as diarrhea and constipation (Mathara et al., 2004). These beneficial effects are possibly related to antimicrobial metabolites, including bacteriocins, as components of such (traditional) fermented commodities. Since the first years of discovery of LAB bacteriocins, numerous have been the subject of intensive investigation (Favaro and Todorov, 2017; Chikindas et al., 2018). According to the current definition, bacteriocins are proteinaceous antimicrobials, produced by bacteria that primarily inhibit the growth of relatively closely-related bacteria; their mode of action is typically bactericidal. It is generally accepted that bacteriocins act by disrupting the cell membrane integrity while different receptors can be involved in this process (Cotter et al., 2013). However, additional mechanisms have also been proposed and may include cleavage of the bacterial DNA, interaction with some intracellular enzymes and/or interrupting bacterial protein synthesis as result of interaction between bacteriocins and ribosome (James et al., 1991; Heu et al., 2001; Todorov et al., 2019). The ability to produce bacteriocins (bacteriocinogenicity) is a common characteristic of many bacteria associated with complex natural ecosystems and may decisively influence the stability of their microbial population. Key focus areas of bacteriocin research have formerly been identified (Schillinger and Holzapfel, 1996), while bacteriocin classification of Gram-positive bacteria has continually developed over a period of more than 20 years (Klaenhammer, 1988; De Vuyst and Vandamme, 1994; Heng et al., 2007). Moreover, it appears that most bacteriocins of Gram-positive bacteria are small peptides, initially expressed as pro-peptides. Only a small number of bacteriocins are posttranslationally modified, as in the case of the lantibiotics (class I in the classification of Klaenhammer, 1988; Heng et al., 2007), while only a few others are relatively complex molecules that also incorporate non-protein moieties responsible for their antibacterial activity (class IV of the classification of Klaenhammer, 1988). Apart from bacteriocins, LAB may produce a variety of antimicrobial compounds, including organic acids, diacetyl, carbon dioxide, hydrogen peroxide, and organic antimicrobial compounds with low molecular mass (Favaro and Todorov, 2017). Particular low-molecular metabolites such as D-phenyllactic and 4-hydroxy-phenyllactic acids, 3-hydroxylated fatty acids and cyclic dipeptides show strong antifungal activity and are therefore of special interest for application in food biopreservation (Valerio et al., 2004; Svanström et al., 2013; Chen et al., 2016; Leyva Salas et al., 2018).

Commercial application of the best studied bacteriocin, nisin, is regulated by the European Food Safety Authority (EFSA) and is licensed as a food preservative (E234). Nisin was recognized as safe for application as a bio-preservative in food products by the Joint Food and Agriculture Organization/World Health Organization (FAO/WHO) Expert Committee on Food Additives in 1969 (Favaro et al., 2015). Subsequently, the FDA approved its use as an “anti-botulism” additive to canned food products in the United States to inhibit *Clostridium botulinum* (Jones et al., 2005). The FAO/WHO Codex Committee on milk and milk products has authorized nisin to be applied as a food additive for different processed cheeses, with an upper limit of 12.5 mg (applied as pure nisin) per kilogram of product (Reis et al., 2012). The exceptionally low cytotoxicity of nisin is comparable to that of NaCl, and it has been safely used in food and food products over a long time period (Jozala et al., 2007). Based on its proteinaceous nature, this may also be attributed to its proteolytic degradation while, at the same time, the sensory attributes of the food remain unchanged (Pongtharangkul and Demirci, 2004).

According to Wiedemann et al. (2001) the underlying mechanism basic to the antibacterial effect of nisin is related to lipid II (the main transporter of peptidoglycan subunits from the cytoplasm to the cell wall) that acts as a docking station (receptor) for nisin. In this way nisin induces the formation of short-lived pores in the cell membrane. The regulation of transport control is thereby disrupted thus leading to the loss of cytoplasmic components (Wiedemann et al., 2001). A similar mode of action has been described for mersacidin and also for the antibiotic vancomycin, both of which bind to a different part of the lipid II molecule (Cotter et al., 2005).

## *Clostridium* and *Clostridiodes* spp.

The enterotoxigenic *Clostridium* and *Clostridiodes* (*Cd*.) spp. can be directly related to food poisoning and non-food−borne human gastrointestinal diseases. Important is their ability to form highly resistant endospores, and, as anaerobes, they are of great concern to both the food canning industry and also to meat producing plants attempting to provide meat products free of *C. perfringens* contamination. *Clostridium* spp. can be transferred to humans and may cause gastrointestinal complications.

The genus *Clostridium* belongs to the family *Clostridiaceae* and, with presently an estimated of more than 203 metabolically diverse species, only a few species are recognized as being pathogenic to humans (NHS, 2016). Representatives are Gram-positive, endospore-forming anaerobic bacteria that are ubiquitously found in virtually all anoxic habitats where organic compounds are present, including soils, aquatic sediments, and the intestinal tracts of animals and humans (Udaondo et al., 2017; Cebrian et al., 2019). By far the majority of the *Clostridium* spp. are considered to be commensal, but a few such as *C. perfringens, C. botulinum, C. tetani*, and *Clostridiodes (Cd.) difficile* are known to be opportunistic, toxin-producing pathogens in both animals and humans and may be associated with a high mortality rate (Cassir et al., 2016; Cebrian et al., 2019; Czepiel et al., 2019). One particular species of the pathogenic ‘clostridia,’ *Cd. difficile*, is receiving increased attention from the clinical community, due to its high resistance to antibiotics and the growing number of especially nosocomial infections with *Cd. difficile* as one of the most common causes (Cebrian et al., 2019; Czepiel et al., 2019). A recent proposal to restrict the genus *Clostridium* to the *C. butyricum* clade and related species resulted in ramifications for “unrelated” *Clostridium* spp. not forming part of this clade (Lawson and Rainey, 2015). With a similarity value of 94.7% between *C. difficile* and *C. mangenotii*, both species were shown to be phylogenetically only distantly related to other members of the genus *Clostridium sensu stricto*. Also by their location within the family *Peptostreptococcaceae* and by their comparatively converging physiological characteristics such as abundant production of H_2_ gas in PYG broth, the reclassification of *C. difficile* and *C. mangenotii* into a new genus, *Clostridiodes*, was proposed by Lawson et al. (2016). For practical purposes the general term “clostridia” in this paper implicitly includes *Clostridiodes difficile*. Confusion still exists related to the simultaneous use of the genus names *Clostridium* and *Clostridioides*. The name *Clostridioides difficile* has been validated in “Validation List No. 171” (Oren and Garrity, 2016), and is acknowledged to meet requirements of the Rules of the International Code of Nomenclature of Prokaryotes (Oren and Rupnik, 2018). According to Oren and Rupnik (2018), the new genus name has not been overruled the use of the name *Clostridium* (yet), which, at present, can still be used equally to *Clostridioides.* We recognize the use of two different genus names (*Clostridium* and *Clostridioides*) in line with the nomenclatural recommendations of the International Committee on Systematics of Prokaryotes of the International Union of Microbiological Societies (IUMS), and accordingly use two different abbreviations (*C*. and *Cd*.) for these two genera.

*Clostridiodes difficile* infections (CDI) are considered as a significant health risk, particularly to an aging population. An estimated 250,000 hospitalizations and 14,000 deaths per year are caused by CDI in the United States alone (Kers et al., 2018), with an estimated annual cost of care amounting to $4.8 billion (Dieterle and Young, 2017). Moreover, recent studies indicate that 30 to 35% of North American CDI cases were due to BI/NAP1/027-type strains (Vaishnavi, 2015; Wilcox, 2016). Relapse following successful antibiotic treatment of CDI, especially when associated with RT027 strains, is not uncommon (Rogers and Whittier, 1928; Surawicz et al., 2013). A major pillar in present-day clinical practices are approaches toward the prevention of infectious diseases. However, current preventative options are limited to antibiotic stewardship and good hygienic practices (Chatterjee et al., 2005; Field et al., 2015). In order to reduce the application of antibiotics, new preventative approaches are urgently needed. An additional and early recognized problem accompanying the use of antibiotics is the elimination or reduction of beneficial microbiota, thereby resulting in dysbiosis and the disruption of gut homeostasis. The unintended loss of diversity and abundance in a stable gut microbiota has a dramatic detrimental impact on colonization resistance of the host to opportunistic pathogens, including *Cd. difficile*, in addition to other negative modulatory influences on gut metabolism (Smith and Hillman, 2008; Ross and Vederas, 2011; Piper et al., 2012; Goetz et al., 2014; Ongey et al., 2017). Discontinuing gut destabilizing antibiotics is considered a first and foremost step in the management of *Cd. difficile* infections (Wilcox and Dave, 2001).

## Bacteriocins and Control of *Clostridium* and *Clostridiodes* spp.

The potential of Class II bacteriocins to modify gut microbiota and to improve host health have been recently reviewed by Umu et al. (2016). In their report the authors have discussed the crossroads between production of bacteriocins and the probiotic potential of LAB for preventing the growth of pathogens in the gut environment.

Antagonistic activity against *Clostridium* and *Clostridiodes* spp. has been reported for diverse bacteriocins representing different Classes, including nisin (Class I), pediocins (Class IIa), plantaricins (Class IIb), different (partly unnamed) enterocins (some belonging to Class IIc), and durancin (Class IV) (Table 1). A bacteriocin is not necessarily active against all tested strains of a species and under all given ecological conditions. A major drawback is the limited knowledge on bacteriocin production and activity in gastro-intestinal tract (GIT) environments *in situ* (e.g., simulation models) or *in vivo* in healthy animals (Umu et al., 2016). The potential of five bacteriocin-producing strains of LAB (producers of sakacin A; pediocin PA-1; enterocins P, Q and L50; plantaricins EF and JK; and garvicin ML), and their isogenic non-producing (bac^–^) mutants were evaluated by Umu et al. (2016) for their probiotic effects by administering the strains to mice through drinking water; changes in the gut microbiota composition were evaluated via appropriate 16S rRNA gene sequencing analysis. Umu et al. (2016) concluded that the overall structure of the gut microbiota remained largely unaffected by the treatments, this also supporting the putative safety of the applied strains. More important observations were only pointing to some lower taxonomic levels. Some potentially problematic bacteria, including *Clostridium* spp., were inhibited when a producer of plantaricins was applied. This can be considered as positive indication that bacteriocin producing strains can be applied for the control of *Clostridium* spp. It appears indeed that they promote favorable changes in the host without major disturbance in the gut microbiota, important for the maintenance of the normal functional properties and gut homeostasis (Umu et al., 2016).

**TABLE 1:** Bacteriocins and their producer strains showing activity against *Clostridium* (*C*.) and *Clostridiodes* (*Cd*.) species with special focus on those associated with infections.

Bacteriocins	Target species*	Experimental set-up	Bacteriocin characteristics	Bacteriocin classification	References
Bacteriocins produced by 6 strains of *E. faecalis*	*C. perfringens*	Vegetative cells	pH stability: 2.0–10.0 thermostability: 60 and 90°C for 30 min; 121°C for 15 min	NR**	Han et al., 2014
Bacteriocin produced by *Lb. plantarum*	*C. butyricum*, *C. difficile*, *C. perfringens*	Vegetative cells	NR	NR	Monteiro et al., 2019
Bacteriocins produced by 2 strains of *St. infantarius* subsp. *infantarius*	*C. perfringens*	Vegetative cells	pH stability: 2.0–12.0 thermostability: 100°C for 120 min; 121°C for 20 min	Class IIa (suggested)	dos Santos et al., 2020
Durancin 61A, produced by *E. durans;* pediocin PA-1 and nisin Z	*Cd. difficile*	Vegetative cells	Durancin 61A: NR pediocin PA-1: NR nisin Z: NR	Durancin 61A: Class IV (glycosylated polypeptide); pediocin PA-1: Class IIa; nisin Z: Class I	Hanchi et al., 2016, 2017
Enterocin AS-48, produced by *E. faecalis*	*Clostridium* spp. (broad spectrum)	Against vegetative cells and spores in food production	Thermostability: heat stable	Class IIc	Egan et al., 2016
Lacticin 481, produced by *L. lactis* subsp. *lactis*	*C. tyrobutyricum*	Cheese production	Thermostability: heat stable	Class I	O’Sullivan et al., 2003
Nisin, produced by *L. lactis*	*C. perfringens*	Endospore germination, vegetative cells	pH stability: 2.0–6.0; thermostability: heat stable	Class I	Udompijitkul et al., 2012
Nisin, produced by *L. lactis*	*Cd. difficile*	Vegetative cells and spores	pH stability: 5.0–9.0; thermostability: heat stable	Class I	Bartoloni et al., 2004; Egan et al., 2016
Nisin, produced by *L. lactis*	*C. tyrobutyricum*, *C. butyricum*, *C. beijerinckii*, *C. sporogenes*	Vegetative cells and spores	pH stability: 5.0–9.0; thermostability: heat stable	Class I	Avila et al., 2014
Nisin, produced by *L. lactis*	*Clostridium* spp.	Cheese production	pH stability: 5.0–9.0; thermostability: heat stable	Class I	Abee et al., 1995; Galvez et al., 2008; Silva et al., 2018
Nisin, lacticin 3147 and thuricin CD, produced by *L. lactis*, *L. lactis* subsp. *lactis* and *B. thuringiensis*	*Cd. difficile*	Vegetative cells	nisin: pH stability: 5.0–9.0; thermostability: heat stable lacticin 3147: pH stability: 5.0–9.0; thermostability: heat stable; thuricin CD: thermostability: heat stable	Nisin: Class I lacticin 3147: Class I	Rea et al., 2007
Plantaricins EF and JK, produced by *Lactobacillus plantarum*	*Clostridium* spp.	Vegetative cells, *in vivo* analysis, animal model	Thermostability: heat stable	Class IIb	Umu et al., 2016
R-type bacteriocin particles; diffocins from *Cd. difficile*	*Cd. difficile*	Vegetative cells	NR	NR	Gebhart et al., 2015; Schwemmlein et al., 2018

* Not all tested strains of a species are intrinsically sensitive to a bacteriocin; ** NR, not reported in the cited reference paper.

Udompijitkul et al. (2012) have evaluated the antimicrobial effect of nisin against two strains of *C. perfringens* (FP and NFB). They have not observed any inhibitory effect against endospore germination of both the food poisoning and non-food−borne strains in a laboratory medium. However, more important was that nisin effectively arrested the outgrowth of germinated spores of *C. perfringens* in a rich medium. Interestingly, Udompijitkul et al. (2012) pointed out that germinated spores of non-food−borne isolates possessed higher resistance to nisin than those of food poisoning strains, pointing to a possible application of nisin in the selective control of *C. perfringens*. Furthermore, nisin exhibited an inhibitory effect against vegetative growth of both FP and NFB isolates in a laboratory medium, with vegetative cells of NFB isolates showing a higher resistance than the FP isolates. Udompijitkul et al. (2012) also reported an inhibitory effect of nisin against both endospore outgrowth and vegetative cells of *C. perfringens* FP and NFB isolates under laboratory conditions, and emphasized the potential of nisin for the control of *C. perfringens*.

Bartoloni et al. (2004) evaluated the effect of nisin activity against clinical isolates of *Cd. difficile* in comparison to the effect of vancomycin and metronidazole based on minimal inhibitory concentrations (MIC), minimum bactericidal concentrations (MBC) and time-kill studies. In this study nisin was found to be more active than the other agents, with a MIC_90_ of 0.256 mg/L and a strong bactericidal activity. Therefore nisin was suggested as a promising agent for the management of *Cd. difficile* associated diarrhea.

The inhibitory influence of several antimicrobial compounds (reuterin, nisin, lysozyme, and sodium nitrite) on both vegetative cells and endospores of *C. tyrobutyricum*, *C. butyricum*, *C. beijerinckii*, and *C. sporogenes* was investigated by Avila et al. (2014) using the experimental protocols of Bartoloni et al. (2004). the effect was compared in two different media, milk and modified RCM (mRCM), after a period of 7 days. Based on the minimal inhibitory concentration (MIC), most *Clostridium* strains showed higher resistance in milk than in mRCM while their endospores were more resistant than the vegetative cells. Vegetative cells and also spores of the investigated clostridia were inhibited in each medium by both reuterin, at MIC values ranging from 0.51–32.5 mM, and nisin at MIC values between 0.05–12.5 μg/mL. Reuterin and nisin, with a broad inhibitory activity spectrum against *Clostridium* spp. spores and vegetative cells, were therefore suggested as the best options to control *Clostridium* growth (Avila et al., 2014).

Several other bacteriocins and antimicrobial proteins from LAB show promise for application in the control of *Clostridium* spp. However, compliance with the safety requirements of the final product should be warranted. Based on the absence of cytotoxicity nisin is considered as safe (Jozala et al., 2007). Moreover, a low cytotoxicity was also reported for several other bacteriocins (Wachsman et al., 2003; Martinez et al., 2013; Carneiro et al., 2014; Quintana et al., 2014; Todorov et al., 2014; Cavicchioli et al., 2018). However, some report have referred to observations on potential cytotoxicity of some bacteriocins, and posed questions on their potential as health hazards (Carneiro et al., 2014; Kaur and Kaur, 2015). However, cytotoxicity of bacteriocins against specific cell lines can be of potential advantage. As Kaur and Kaur (2015) have pointed out, high (selective) bacteriocin cytotoxicity against cancer cells but without similar action against healthy cells, can be explored as an advantage in the control and treatment of some types of cancers.

The safety of bacteriocin producers may constitute an additional concern. Generally the LAB are recognized as safe, and most species have GRAS status. As a large group of Gram-positive, non-sporeforming, catalase-negative, fermentative and generally aerotolerant bacteria, the LAB include diverse organisms, adapted to specific and partly extreme environments, and with some (most *Streptococcus* spp.) considered as typical pathogenic. On the other hand, clinical cases of *Lactobacillus* associated bacteremia and sepsis have been reported, yet, only in rare cases and in particular for patients with underlying conditions (Castro-González et al., 2019). Therefore, any declaration on safety requires critical assessment and careful specification, also taking into account both general phenotypic heterogeneity and subspecies diversity. Some *Enterococcus* species are considered as opportunists and are frequently associated with nosocomial infections (Holzapfel et al., 2018); strains from clinical origin are known to carry virulence factors (Favaro et al., 2014; Perin et al., 2014; Ribeiro et al., 2014). With almost all species of the genus *Streptococcus* considered as pathogenic, *Streptococcus thermophilus* represents a rare exception with GRAS status, and with relatively strict specialization in the dairy environment (Leuschner et al., 2010; dos Santos et al., 2020). Some recent papers have reported on the safety of *Streptococcus diacetylactis* subps. *macedonicus* and suggested its safe application as starter and probiotic culture (De Vuyst and Tsakalidou, 2008; Pieterse et al., 2010; Laiño et al., 2019).

*Streptococcus infantarius* subsp. *infantarius* is reported to be the predominant species in several dairy products in West Africa and Brazil (Schlegel et al., 2000; Hoshino et al., 2005; Abdelgadir et al., 2008; Herrera et al., 2009; dos Santos et al., 2020). Interestingly, bacteriocin production have been reported for two strains of *St. infantarius* subsp. *infantarius*, isolated from the dairy environment, showing a strong activity not only against *Listeria monocytogenes*, but against *C. perfringens* (dos Santos et al., 2020). Such activity suggests the potential of the producer strains (*St. infantarius* subsp. *infantarius* K1-4 and K5-1) as candidates for application as starter cultures, and even as putative probiotics. dos Santos et al. (2020) explored different characteristics and thee physiological behavior of *St. infantarius* subsp. *infantarius* strains K1-4 and K5-1 and, while suggesting their potential as beneficial strains, also mentioned the detection of several virulence factors. Declaring these strains as safe for human and animal applications therefore appears premature. This report (dos Santos et al., 2020) serves as a good example for the importance of safety evaluation of each particular strain before recommending it for use as beneficial culture. On the other hand, biotechnological production of a bacteriocin of even an unsafe strain may be feasible in view of the appropriate application of the purified bacteriocin as antimicrobial agent.

Han et al. (2014) reported on bacteriocin-producing *Enterococcus faecalis* strains with activity against *C. perfringens*, isolated from domestic animals an with a potential for application as probiotics. From a total of 1370 evaluated bacterial isolates, 6 were selected on basis of their activity against the pathogenic indicator strains *C. perfringens* KCTC 3269 and *C. perfringens* KCTC 5100. All selected 6 isolates were identified as *E. faecalis* by 16S rRNA sequencing. Apart from their anti-*Clostridium* activity, selected strains showed potential as probiotics based on their behavior under *in vitro* gut model conditions. Some of the evaluated *E. faecalis* strains also showed strong inhibitory activity against different strains of *Listeria monocytogenes*. The produced bacteriocins were partially characterized, and Han et al. (2014) suggested the use of these bacteriocin producing strains and/or their bacteriocins in feed manufacturing in the livestock industry as alternatives to antibiotics.

The *in vitro* antimicrobial activity and potential probiotic application of *Bifidobacterium* and *Lactobacillus* against *Clostridium* species were investigated by Monteiro et al. (2019). Based on its inhibitory activity the authors suggested the application of *Lb. plantarum* ATCC 8014 as promising candidate against *Clostridium* spp. In addition, this strain may be considered as probiotic based on its functional properties. Referring to its potential for controlling infections by *Clostridium* species, Monteiro et al. (2019) suggested safety evaluation of *Lb. plantarum* ATCC 8014 in *in vivo* animal models in order to clarify open questions before its application as beneficial organism.

Accumulating knowledge on the importance of human gut microbiota diversity suggests that reduction in microbial diversity may result in vulnerability to many diseases (Vujkovic-Cvijin et al., 2013; Gevers et al., 2014; Xuan et al., 2014; Gebhart et al., 2015). A definitive link between human gut microbial diversity, gut homeostasis and health appears to be firmly established. Even when acknowledging the extraordinary importance of conventional antibiotics, the other side is pointing to negative consequences resulting from the inadverse antibiotic application for (sometimes extended) treatment of bacterial diseases, also resulting in off-target effects (Gebhart et al., 2015). There is a clear need for more selective and highly effective, so-called “smart,” antibacterial agents by which rapid and accurate molecular diagnostic information can be exploited at the point of intervention toward precisely targeted protection against a pathogen. Such new generation antimicrobials pose a low risk of drug resistance transfer to off-target organisms or disrupting gut homeostasis by the elimination of protective/beneficial microbiota. Precision antibacterials can be deployed both as safe prophylactics and therapeutics. Safe antibacterials such as bacteriocins, combinations of bacteriocins and selected drugs, and bioengineered antimicrobial or chemically modified peptides may be considered as the next frontier in the combat of humans and animals against pathogens (Gebhart et al., 2015).

Antibiotic resistance is widely known and well described for, e.g., methicillin-resistant *Staphylococcus aureus* (MRSA) and *Cd. difficile*. There is wide consensus on an urgent need for alternatives to antibiotics and for finding novel antimicrobial compounds to combat these pathogens (Allen, 2017). In the last decade a strong increase in outbreaks of *Cd. difficile*-associated disease (CDAD) were registered. These presented significant challenges to health care facilities worldwide (Dieterle and Young, 2017). *Cd. difficile* was recognized as a causative agent of nosocomial diarrhea since the early 1970s (George et al., 1978; Rea et al., 2010). Moreover, it is generally acknowledged that CDAD is increasing, not only quantitatively, but also in severity in many parts of the world (Redelings et al., 2007). The scientific community accepts the fact that the main predisposing factor for CDAD is antibiotic therapy, which often eradicates the commensal and beneficial gut microbiota of the host and enables the opportunistic establishment of a *Cd. difficile* infection. Several antibiotics, hitherto considered as effective therapeutic agents, have been implicated in CDAD, and include clindamycin, ampicillin, and amoxicillin as well as the cephalosporins and fluoroquinolones (Aronsson et al., 1985; Wiström et al., 2001; Bartlett, 2006). However, their negative effects on the commensal gut microbiota are recognized, while the increase in antibiotic resistance constitutes an additional problem. Moreover, the increasing prevalence of hypervirulent strains of *Cd. difficile* (Bartlett, 2006; Kuijper et al., 2007) adds urgency to the search for alternative treatment of CDAD. Thus far, small-molecule antibiotics have generally proven unsuccessful in mitigating the development of resistance in bacterial pathogens, indicating a need to examine alternative classes of antimicrobial compounds. Linked to this, there has been considerable interest in bacteriocins as antimicrobial bacterial peptides with either narrow- or broad-spectrum antimicrobial activities. It has already been established that bacteriocins such as nisin and lacticin 3147 can effectively kill *Cd. difficile* at concentrations that compare favorably with therapeutic levels of vancomycin and metronidazole, the most commonly used antibiotics in the treatment of CDAD (Rea et al., 2007). Moreover, thuricin CD, a two-component antimicrobial showed activity against *Cd. difficile* in the nanomolar range. This antimicrobial peptide (thuricin CD) is produced by *Bacillus thuringiensis* DPC 6431, a strain originally isolated from a human fecal sample, and consisting of two distinct peptides, Trn-α and Trn-β, that act synergistically to kill a wide range of clinical *Cd. difficile* isolates, including ribotypes commonly associated with CDAD (Rea et al., 2010). Very important is the fact that thuricin CD has little impact on most other genera, including many gut commensal microorganisms. The amino acid sequences of thuricin SD molecules were determined using infusion tandem mass spectrometry and revealing that each peptide is posttranslationally modified at its respective 21st, 25th, and 28th residues. The thuricin CD gene cluster contains genes responsible for encoding two S′-adenosylmethionine proteins that are typically involved in uncommon posttranslational modifications. The two components of the thuricin CD antimicrobial peptide system are connected by sulfur to α-carbon linkages. It shows prospects for therapeutic implementation targeted against CDAD, with the additional benefit of not disturbing normal gut microbial homeostasis (Rea et al., 2010).

Some strains of *Cd. difficile* produce phage tail-like particles upon induction of the SOS response. These particles have bactericidal activity against several *Cd. difficile* strains and can therefore be classified as bacteriocins, similar to the R-type pyocins of *Pseudomonas aeruginosa*. Each of these R-type bacteriocin particles, purified from different strains, shows a different *C. difficile*-killing spectrum, with no single bacteriocin killing all *C. difficile* isolates tested. The genetic locus has been identified for these “diffocins” and was found to be common within the species (Schwemmlein et al., 2018). Moreover, the entire diffocin genetic locus of more than 20 kb was cloned and expressed in *Bacillus subtilis*, resulting in the production of bactericidal particles. One of the interesting features of these particles is a very large structural protein of 200 kDa. It determines the lethal spectrum of the particles and is likely the receptor-binding protein. Diffocins may provide an alternative bactericidal agent to prevent or treat infections and to decolonize individuals that are asymptomatic carriers (Schwemmlein et al., 2018).

Some specific bacteriocin-like metabolites such as high-molecular-weight or phage tail-like bacteriocins appear to be common throughout the Eubacteria domain (Gebhart et al., 2012). From this family of antimicrobials, the best-studied examples are possibly the R-type pyocins produced by some strains of *P. aeruginosa* (Michel-Briand and Baysse, 2002); these have in fact been well reported earlier for other Gram-negative (Coetzee et al., 1968; Strauch et al., 2001) and also Gram-positive bacteria (Kautter et al., 1966; Ellison and Kautter, 1970; Thompson and Pattee, 1981; Zink et al., 1995; Gebhart et al., 2012). These phage tail-like bacteriocins are produced as intracellular metabolites in response to SOS induction and are liberated only after cell lysis. Gebhart et al. (2012) subdivided phage tail-like bacteriocins in R and F-types, of which the R-type resembles the structures of the tail apparatus of the Myoviridae phages (contractile, non-flexible tails), while the F-type is described for *P. aeruginosa* with genetic resemblance to the Siphoviridae phages (non-contractile, flexible tails) (Nakayama et al., 2000). In their mode of action, R-type bacteriocins kill target cells by attachment through interaction between a receptor binding protein (tail fiber protein) and a bacterial surface receptor. This leads to insertion of the core through the envelope of the target bacterium and rapid depolarization of the cell membrane potential and immediate cell death (Uratani and Hoshino, 1984). The activity spectrum of R-type bacteriocins generally includes very closely related species, normally other strains from the same species, although rare coincidental killing of other species by R-type pyocins has been noted; moreover, autoimmune phenomena have been reported (Gebhart et al., 2012). R-type phage tail-like bacteriocins (“diffocins”) can be mapped in the genomes of different strains of *Cd. difficile*. Some strains of this species can produce diffocin particles as a result of SOS response induction; these strains may show bactericidal effects against other *Cd. difficile* strains (Gebhart et al., 2012).

Biotechnical application of “diffocin” was proposed by Gebhart et al. (2015) in designing a specific genetically modified contractile R-type bacteriocin from *Cd. difficile* strain CD4 with the purpose to kill BI/NAP1/027-type strains. Gebhart et al. (2015) proposed that the natural receptor binding protein (RBP) responsible for diffocin targeting can be replaced by a RBP identified protein from the prophage of a BI/NAP1/027-type target strain. The resulting modified diffocins, called avidocin-CD type Av-CD291.1 and Av-CD291.2, respectively, were reported to be more stable and to kill the 16 BI/NAP1/027-type strains (applied as target strains) investigated. In addition, Gebhart et al. (2015) evaluated the efficacy of Av-CD291.2 in an animal model, providing the constructed diffocin via the drinking water. Sufficient survival levels during the passage through the mouse GIT were reported, yet, not notably modulating the mouse gut microbiota or even disrupting natural colonization resistance to *Cd. difficile* or a vancomycin-resistant *E. faecium* (VREF) strain. Moreover, antibiotic-induced colonization of the mice inoculated with BI/NAP1/027-type *Cd. difficile* spores was prevented (Gebhart et al., 2015), thereby reducing presence of the most severe CDIs. In conclusion, this modified diffocin can be considered as a prototype of an “Avidocin-CD platform,” for producing targetable agents with high precision and efficacy against *Cd. difficile*. This represents a basis for the prevention and combating of CDIs without a negative impact on commensal (protective) indigenous microbiota (Gebhart et al., 2015).

## Bacteriocins, Cheese Preservation and Control/Prevention of *Clostridium*

Control of clostridia is a challenging issue, not only in human and veterinary medicine, but also a serious problem in the dairy industry. Bacterial contamination of dairy products with *L. monocytogenes*, some *Staphylococcus* spp., and/or sporeformers such as *Bacillus* and *Clostridium* spp. is considered as a potential health hazard. Nisin has been confirmed as effective antimicrobial agent for the control of numerous Gram-positive bacteria, including spoilage LAB and pathogens (such as *L. monocytogenes*, *S. aureus*, some *Bacillus* spp., and *Clostridium* spp.) (Silva et al., 2018). Nisin application to prevent late blowing in cheese caused by gas-producing *Clostridium* spp. was widely explored and applied by the dairy industry (Galvez et al., 2008). Nisin also finds application in cheeses and pasteurized cheese spreads as alternative to nitrate for preventing the outgrowth of *Clostridium* spores (Abee et al., 1995; Silva et al., 2018).

Another lantibiotic, lacticin 481, was also suggested as an alternative for the control of *Clostridium tyrobutyricum* (O’Sullivan et al., 2003) and *L. monocytogenes* (Ribeiro et al., 2016). The advantage of lactacin 481 is its specific spectrum of activity, also by generally being not active against other LAB, thus rendering it a good candidate for use in fermented food products (Silva et al., 2018). Moreover, a mild bacteriostatic activity has been shown for non-purified lacticin 481 in milk stored at refrigeration temperatures (Arqués et al., 2011). Also, semi-purified lacticin 481 can be applied with high success to fresh cheeses stored at refrigeration temperatures in reducing *L. monocytogenes* viable numbers by 3 log cycles within 3–7 days (Ribeiro et al., 2016).

Enterocin AS-48, a cyclic bacteriocin, produced by *E. faecalis* is active against a number of *Bacillus* and *Clostridium* strains (Egan et al., 2016). The activity of several enterocins against foodborne pathogens, including *Listeria* spp. and *Clostridium* spp., has been well established Yet, their application in food systems needs more precise evaluation. This especially includes their optimization, e.g., by addition of possible synergistic components for improved prevention of the re-growth of pathogens throughout storage (Khan et al., 2010; Zacharof and Lovitt, 2012; Barbosa et al., 2015a,b). A possible hypothesis maybe related to insufficient activity of these bacteriocins against *Clostridium* endospores.

Apart from nisin producing strains, other bacteriocin-producing LAB have also been proposed as alternatives for the prevention of *Clostridium* associated late blowing of cheeses, commonly related to the ubiquitous presence of *Clostridium* spores in the dairy environment (Gómez-Torres et al., 2015). It could be that one single alternative for reducing germination of *Clostridium* spores might not be effective to prevent late blowing in cheeses (Garde et al., 2011). Approaches involving combined factors therefore deserve further investigation, and may include bacteriocins and/or bacteriocinogenic LAB as alternative starters or adjustment cultures for cheese-making.

## Bacteriocin Synergism Studies and Delivery Systems

There appears to be promising scope for the application of different non-antibiotic antimicrobial agents such as bacteriocins for controlling pathogenic microbes in therapeutic practices. This approach needs to be viewed as part of a potentially complex treatment procedure that may also incorporate synergies with conventional antimicrobial agents. Some studies have already pointed to benefits of synergistic interactions between bacteriocins and antibiotics with the clear objective of reducing the usage of antibiotics and other conventional drugs, and also of increasing their efficacy (Minahk et al., 2004; Todorov and Dicks, 2009; Salvucci et al., 2010; Todorov et al., 2010; Todorov, 2010). To our best of knowledge, only limited studies have dealt with the evaluation of combined applications and for exploring synergistic mode(s) of action of bacteriocins and antibiotics against *Clostridium* spp. (Mathur et al., 2013; Hanchi et al., 2017). However, activity against *Listeria* spp. has been well documented and the anti-*Listeria* efficacy of enterocin CRL35 was shown when combining with the cell wall membrane-acting antibiotics (monensin, bacitracin, and gramicidin) and muralytic enzymes (mutanolysin and lysozyme) (Salvucci et al., 2010). A number of benefits from this combined application, positive interactions and possible synergistic effects were reported. According to Salvucci et al. (2010) the combination of not only the naturally produced bacteriocin, enterocin CRL35, but also of its synthetic equivalent with some antibiotics can provide substantial inhibitory effects against *L. innocua* and *L. monocytogenes* growth in culture; even more importantly, this combination can significantly reduce the amount of antibiotic required for effective treatment of infections caused by *Listeria* spp. Enterocin CRL35 appears to be a promising agent with applications not only for maintenance of food quality and safety, but also for medical applications in combination with conventional therapeutic agents. The former examples suggest a therapeutic potential of antibiotic and bacteriocin combinations by which the risk of the possible misuse of antibiotics in human and veterinary medicine can be reduced. This may also be a precaution against the development of antibiotic-resistant strains.

Actagardine, the lipid II-binding lantibiotic, was applied in combination with the antibiotics metronidazole, vancomycin and ramoplanin in an assay against several *Cd. difficile* isolates (Mathur et al., 2013). In combination with ramoplanin actagardine behaved in a partial synergistic/additive fashion against 61.5% of the target *Cd. difficile* strains assessed in this study (Mathur et al., 2013). Actagardine-metronidazole and actagardine-vancomycin combinations also showed partial synergistic/additive effects against 54 and 38% of *Cd. difficile* strains, respectively (Mathur et al., 2013).

Combinations of durancin 61A with reuterin or with pediocin showed strong synergistic effects against *Cd. difficile*. With this approach the MIC of *Cd. difficile* could be reduced from 16 mg/L to respectively 0.12 (in presence of reuterin) and 0.06 mg/L (with pediocin) (Hanchi et al., 2017). Durancin 61A, a glycosylated broad-spectrum bacteriocin produced by *E. durans* 61A, was active against clinical drug-resistant *C. difficile*, in addition to *E. faecium* vancomycin- resistant enterococcus (VRE) and MRSA. Synergistic inhibition of *Cd. difficile* was also detected when durancin 61A was combined with reuterin and pediocin (Hanchi et al., 2017). Durancin 61A was shown to be bactericidal and to act on the bacterial membrane via the pore formation mechanism that includes pore and vesicle formation followed by cell disruption and loss of membrane integrity and cytoplasm content (Hanchi et al., 2016). Combinations of durancin 61A with antibiotics and other bacteriocins showed highly synergistic inhibitory patterns against multi-resistant pathogens related to clinical cases, including *S. aureus, Cd. difficile* and *Streptococcus* spp. (Hanchi et al., 2017).

Although bacteriocins have clearly been reported in recent years to show promise as therapeutic antimicrobial agents, the development of appropriate delivery systems remains a considerable challenge for successful therapeutic applications. In an overview on existing bacteriocin delivery systems, Arthur et al. (2014) referred to their potential for application both in food bio-preservation and in human and veterinary medicine. Several promising delivery systems for bacteriocins have been suggested such as silver- or carbohydrate-based nanoparticles, nanofiber scaffolds, nanospheres, impregnated implants, catheter coating, hydrogel, oral tablets, gums, livestock feed, aquaculture dry spray, and also the incorporation in food packaging, and of either the bacteriocin or its producer strain in food products (Favaro et al., 2015).

## Phages in Combat Against *Clostridium* spp.

Naturally occurring bacteriophages (phages) appears to be an interesting alternative option for therapeutic treatment of infected livestock, even before conventional antibiotics have been used in farming practice for this purpose (Gravitz, 2012; Ghosh and Kim, 2019). In general, bacteriophages can act bactericidal by anchoring onto the bacterial cell surface, followed by injection of phage genetic material into the bacterial cytoplasm. This results in the take-over of the host cell machinery and the synthesis of phage components, followed by the assembly of new phages within the infected bacterial cell. In most cases, this leads to bacterial lysis and the release of phage progeny that can commence a second infection cycle. Phages are known to be highly selective in target (host) selection and therefore will attack only specific bacterial species or even strains, according to their mode of selectivity. This is a well-know principle and highly promising for exploitation of the lytic cycle of bacteriophages.

Further development that may lead to practical application of phage therapy for selectively targeting pathogenic bacteria over commensal bacteria is justified (Clark and March, 2006). However, despite the successful use of phage therapy in Eastern European countries and especially in the former Soviet Republics, the Western world previously failed to follow up on the development of phage therapy (Pelfrene et al., 2016). Currently, phage therapy is the subject of increased research, and is driven by emerging needs for alternatives to traditional antibiotics (Kutter et al., 2010; Abedon et al., 2011). Bacteriophages present promising alternatives for the treatment of various bacterial infections, including *Cd. difficile.*

Even when phage therapy offers several advantages, some concerns exist about its practical application. Bacterial resistance to homologous phages has been reported (Labrie et al., 2010), and assessing the susceptibility of bacteria to a particular phage is therefore crucial. Unfortunately, rapid diagnostic platforms are not yet available, thereby frequently requiring a cocktail of multiple bacteriophages for the treatment of selected pathogen/s. Another problem may be the release of endotoxins from the bacteria after bacteriophage lysis; this could potentially lead to sepsis. Moreover, the pharmacokinetics of bacteriophages is a concern as they are known to easily diffuse into several organs of the body (Abedon et al., 2011). A possibly more serious concern is the immunogenicity of bacteriophages (Abedon et al., 2011). These aspects may limit to the uses of bacteriophages beyond a single application, as they would be efficiently cleared by the body the next time they are administered. Still, advances in genetic engineering during the recent decades have ensured that bacteriophages can be used in innovative ways to combat bacterial infections (Braff et al., 2016). On the other hand, inadequate methodology for phage preparation may constitute a major hurdle toward their development for successful therapy. The presence of endotoxins and pyrogenic substances poses a high degree of toxicity potential, and their removal would therefore be essential for safe application (Ghosh and Kim, 2019).

## Probiotics in Combat Against *Clostridiodes difficile*

Different probiotics have been suggested over a long period as potential and effective agents for prevention and control of diarrhea. The potential of probiotics such as *Saccharomyces boulardii* in biotherapeutic approaches for restoration of gut microbiota and combating *Cd. difficile* associated infections has already been recommended earlier (Buts et al., 1993; Rolfe, 2000; Wilcox and Dave, 2001; Aslam et al., 2005).

The clinical characteristics of diarrhea can be very different, but this condition is generally associated with a disbalance (dysbiosis) in the GIT microbiota; it frequently results from (inadverse) antibiotic treatment. *Cd. difficile* is typically involved in nosocomial antibiotic-associated diarrhea and is also considered to be the major agent in the etiology of pseudomembranous colitis (Hopkins and Macfarlane, 2003). Active drug therapy of diarrhea normally involves antibiotic treatment, also based on the expected presence of pathogenic bacteria involved in the production of toxins, thereby resulting in the disruption of gut homeostasis. Such therapies can, in some cases, even provoke life-threatening conditions for a patient and is also related to the possible destruction of both beneficial and commensal GIT microbiota by antibiotics. The use of probiotics in such cases can be considered as superior and potentially more effective alternative to antibiotics, also because the specificity of probiotics in targeting some specific pathogens constitutes a well-documented advantage. In addition, probiotics can be applied as a prevention therapy by not altering the beneficial microbiota and rather supporting the stabilization the gut microbiome.

Recommendations of the European Society of Clinical Microbiology and Infectious Diseases (ESCMID) for treatment of *Cd. difficile* infections include therapy with antibiotics, toxin-binding resins and polymers, immunotherapy, probiotics and fecal or bacterial intestinal transplantation (Debast et al., 2014). Generally, antibiotic treatment is a first choice in clinical practice and includes the application of metronidazole, vancomycin and fidaxomicin (Debast et al., 2014). However, antibiotic resistance and collateral effects of overdosing antibiotic applications are reasons for searching alternative therapies including the application of probiotics, live therapeutics and even bacteriocins such as thuricin CD. These were suggested as effective alternatives to reduce incidences and recurring infections (Rea et al., 2013).

Bacteriocins represent viable alternatives to antimicrobials due to their specificity, related to either a narrow or broad spectrum of antimicrobial activity. In addition, the potential of bacteriocins is also underlined by their low toxicity, the possibility of their *in situ* production by probiotic organisms, and their modulatory potential for bioengineering (Mathur et al., 2014). However, as potential alternative bacteriocin therapy for specifically combating *Cd. difficile* infections, their comparative effectivity relative to currently used antibiotics needs to proven while the risk of damage to the GIT microbiota should be excluded. Nisin and lacticin 3147 are bacteriocins showing efficacy in killing *Cd. difficile in vitro* at concentrations comparable to those used in the application of vancomycin and metronidazole (Mathur et al., 2014). Thuricin CD, a two-component peptide shows potential in the treatment of *Cd. difficile* infections (Rea et al., 2010; Mathur et al., 2014). Mathur et al. (2014) reported on GE2270 and its semi-synthetic derivative LFF571 as two additional bacteriocins with antimicrobial activity against *Cd. difficile*. Trzasko et al. (2012) conducted a pre-clinical study on survival benefits of peptide LFF571 compared to vancomycin at a lower dose, resulting in fewer recurrences in the hamster *Cd. difficile* infection model. In addition, the safety, tolerability and pharmacokinetics of single and multiple ascending oral doses of LFF571 were investigated. Trzasko et al. (2012) further reported that among 56 subjects, protein LFF571 was well tolerated and no serious side-effects were noted. A low concentration of protein LFF571 remained in the serum compared to a high concentration in the fascia. These results suggest the scope for the future development of protein LFF571 as potential agent for treatment for CDI (Trzasko et al., 2012).

Strategies based on the (beneficial) modulation of gut microbiota including the application of life microorganisms, are strongly pointing to the potential of alternative (biological) approaches for the control of CDI. The use of probiotics and prebiotics (non-digestible oligosaccharides) constitutes two of several alternatives for successfully preventing CDI and antibiotic-associated diarrhea in children (Hopkins and Macfarlane, 2003). Especially in the case of infants and young children, only carefully selected probiotic strains should find specified clinical application (Zheng et al., 2017), also by considering the strain related functionality (McFarland, 2005) and safety (Brodmann et al., 2017) of a probiotic. Moreover, current developments in both precision medicine and precision nutrition are taking into account the diversity patterns of the human gut microbiome (Jobin, 2018; Mills et al., 2019). Individual differences in the commensal microbiota and genes of the human gut indeed constitute a strong basis for the rapid development of personalized disease management toward so-called “microbiomics” (Shukla et al., 2015). Detecting changes in the relative abundance of particular gut microbiota may serve as a strong diagnostic tool for analyzing health conditions including a variety of gut related diseases (Kitsios et al., 2016; Almonacid et al., 2017). On this basis the association of *Cd. difficile* with diarrhea could be clearly identified. In fact, individualized host–microbiome phenotypes are reflected by the maintenance of host–microbe associations across populations, thereby constituting an essential key for precision medicine (Petrosino, 2018). In a wider sense, tungstate treatment for modulating (“precision editing”) the gut microbial population has been suggested for ameliorating dysbiotic conditions such as colitis (Zhu et al., 2018).

Using a co-culture method, Quigley et al. (2019) proposed the use of different *Lactobacillus* strains for the control of *Cd. difficile* activity. Following preliminary screening for inhibitory ability, the efficacy of *Lb. gasseri* APC 678 and *Lb. rhamnosus* DPC 6111 to combat *Cd. difficile* was shown in a murine model of CDI (Quigley et al., 2019). The beneficial effects of *Lb. gasseri* APC 678 was confirmed by the significant reduction of viable *Cd. difficile* VPI 10463 numbers in the feces of mice. Moreover, additional analysis based on the sequencing of the cecal microbiota showed that application of *Lb. gassei* APC 678 in a mouse model resulted in a significant increase in bacterial diversity (Quigley et al., 2019). At the same time, application of *Lb. gassei* APC 678 did not significantly affect the relative abundance of Firmicutes or Bacteroidetes, relative to the control.

The hypothesis related to strain specific benefits of probiotics was discussed and defended in different cases (Quigley et al., 2019). Consequently Quigley et al. (2019) strongly defended the idea of strain specificity, evaluating the range of *Lactobacillus* species for their efficacy to inhibit *Cd. difficile* with the aim to select an appropriate potential strain/s to target CDI in humans. Based on these results Quigley et al. (2019) highlighted the potential of *Lb. gasseri* APC 678 as a live therapeutic agent for targeting CDI.

In a micro-calometric study, Fredua-Agyeman et al. (2017) evaluated the *in vitro* inhibition of *Cd. difficile* by commercial probiotic strains and mixtures (*Lb. acidophilus* LA-51, *B. lactis* BB-121, Probio 71, and Symprove^TM^). Li et al. (2019) reported on the successful attenuation of *Cd. difficile* colonization by a probiotic consortium of five *Lactobacillus* and two *Bifidobacterium* strains in a mouse model, and proposed the modulation of gut microbiota and bile acids as the underlying mechanism for this effect.

*Saccharomyces boulardii* is a probiotic yeast that can upregulate the expression of anti-toxin A secretory IgA and that secretes a protease that degrades toxin A and B produced by *Cd. difficile* and, in this way, effectively contributes to the control of CDI (Hickson, 2011). McFarland (2005) reported on a double-blind placebo-controlled trial, performed with high doses of vancomycin in combination with *S. boulardii*, and detected a significant reduction in recurrence of CDI compared to patients receiving only a high dose vancomycin and placebo patients.

## Final Remarks

In a report of 31 July 2020^1^, the World Health Organization (WHO) describes the current antibiotic resistance situation as “one of the biggest treats to global health, food security and development.” Multiple factors are underlying to the emerging rise of antibiotic resistance, with over- or mal-prescription, and extended (and wrong) use as some of the major complicating contributors, both in human medicine and animal husbandry. Looking for therapeutic alternatives to classical antibiotics constitutes an enormous but rewarding challenge. Thanks to a better understanding of the underlying antibiotic resistance mechanisms, and the access to information on the whole genome of resistant pathogens, a rapidly widening spectrum of potential antimicrobial agents has become a focus of current research. With this paper we intended to highlight the antimicrobial peptides, including bacteriocins, as a highly promising group of biotherapeutics, as alternatives to conventional antibiotics, for the treatment and prevention, especially of *Clostridium* infections. Several bacteriocins have already displayed efficacy in the laboratory and pre-clinical experiments. Some bacteriocins, and in particular nisin, have found successful commercial application in the food industry. We expect that *in vivo* (and in part also clinical) studies will be imperative before bacteriocins can be more regularly integrated in approaches for the control of bacterial and viral infections in human and veterinary medicine.

## Author Contributions

WH and ST planned and developed the manuscript. H-JK and II contributed to further expanding the text. ST contributed to the semifinal version, while WH added further amendments and did the final editing. All authors contributed to the article and approved the submitted version.

## Conflict of Interest

H-JK and WH are employed by company HEM Inc. The remaining authors declare that the research was conducted in the absence of any commercial or financial relationships that could be construed as a potential conflict of interest.

## Footnotes

1 https://www.who.int/news-room/fact-sheets/detail/antibiotic-resistance
